# The Physiological Significance of A-Waves in Early Diabetic Neuropathy: Assessment of Motor Nerve Fibers by Neurophysiological Techniques

**DOI:** 10.3389/fnsys.2021.633915

**Published:** 2021-01-28

**Authors:** Qiong Cai, Guliqiemu Aimair, Wen-Xiao Xu, Pei-Yao Xiao, Lie-Hua Liu, Yin-Xing Liang, Chao Wu, Song-Jie Liao

**Affiliations:** ^1^Department of Neurology, The First Affiliated Hospital, Sun Yat-sen University, Guangzhou, China; ^2^Guangdong Provincial Key Laboratory of Diagnosis and Treatment of Major Neurological Diseases, National Key Clinical Department and Key Discipline of Neurology, Guangzhou, China; ^3^Department of Endocrinology and Diabetes Center, The First Affiliated Hospital, Sun Yat-Sen University, Guangzhou, China

**Keywords:** type II diabetes, diabetic neuropathy, A-waves, conduction velocity distribution, nerve conduction studies

## Abstract

**Objective:** This study aimed to investigate how early A-waves could occur in type II diabetes, and what it implied functionally.

**Methods:** We performed conduction velocity distribution (CVD) test in peroneal nerves of 37 type II diabetic patients with normal nerve conduction study (NCS) and 22 age-matched controls. The electrophysiological data and clinical information were analyzed.

**Results:** A-waves were observed in 45.9% of diabetic patients and only in 1 person in healthy controls, all detected in the tibial nerves. The diabetic patients with A-waves showed faster conduction velocity in all quartiles in the motor peroneal nerves compared to the patients without A-waves, and their CVD histograms were shifted to the right side, consisting of a significantly larger percentage of fast conducting fibers. There was no significant difference in the CVD values of the upper extremity nerves among the patients with and without A-waves and the healthy controls.

**Conclusion:** A-waves could occur in type II diabetes as early as when NCS showed normal, and represented as a sign of neuropathy as well as a sign of rescued motor nerve function.

## Introduction

A-wave is a late response following the compound muscle action potential (CMAP, M-wave) during routine motor nerve conduction studies (NCS). The term has been used in a more general manner for abnormal late responses recorded in F-wave studies with supramaximal stimuli, and A-wave is distinguished from F-wave by a constant shape and latency (Rowin and Meriggioli, 2000; Jerath and Kimura, 2019). A-waves were proposed to be an indicator of underlying neuropathy, frequently found in many neurogenic disorders, including demyelinating neuropathies, axonal neuropathies, radiculopathies, and motor neuron diseases (Kawakami et al., 2012; Fang et al., 2017). Very lately, A-waves were reported to have an increased incidence in patients with distal diabetic peripheral neuropathy (DPN) (Rampello et al., 2019), however, its physiological significance was not yet clarified and more advanced electrophysiological studies should be conducted.

NCS, the best approach till date for measuring peripheral nerve function in DPN due to its accuracy, reliability and sensitivity, preferentially carries information for the nerve fibers with the largest axon diameters and the fastest conduction velocities, thus underestimates early DPN (Tesfaye et al., 2010). But the analysis of F-waves increased the sensitivity of NCS (Pan et al., 2014). In addition, the method to calculate the contribution of each nerve fiber group of different conduction velocities that makes up the compound muscle action potential, so called the conduction velocity distribution (CVD), is an accurate way to detect nerve damages and more sensitive for early diabetic neuropathy compared to routine NCS (Tuncer et al., 2011; Koszewicz et al., 2019). CVD can detect the loss of motor fibers with different conductive velocities in diabetic patients when NCS remains normal, thus can serve as a supplement for routine NCS in early DPN (Kiziltan et al., 2007; Tuncer et al., 2011).

Herein this study, we aimed to investigate the significance of A-waves in early diabetic neuropathy. We identified type II diabetic patients whose NCS was in normal range by using standard conduction techniques, and analyzed CVD of motor nerves in order to find the neurophysiological significance of A-waves in early DPN.

## Materials and Methods

### Ethics Statement

Ethics approval was obtained by the ethics board of the Ethics Committee of the First Affiliated Hospital of Sun Yat-Sen University, Guangdong, China. The study was carried out only after written informed consent was obtained from all participants.

### Subjects

**Figure 1:**
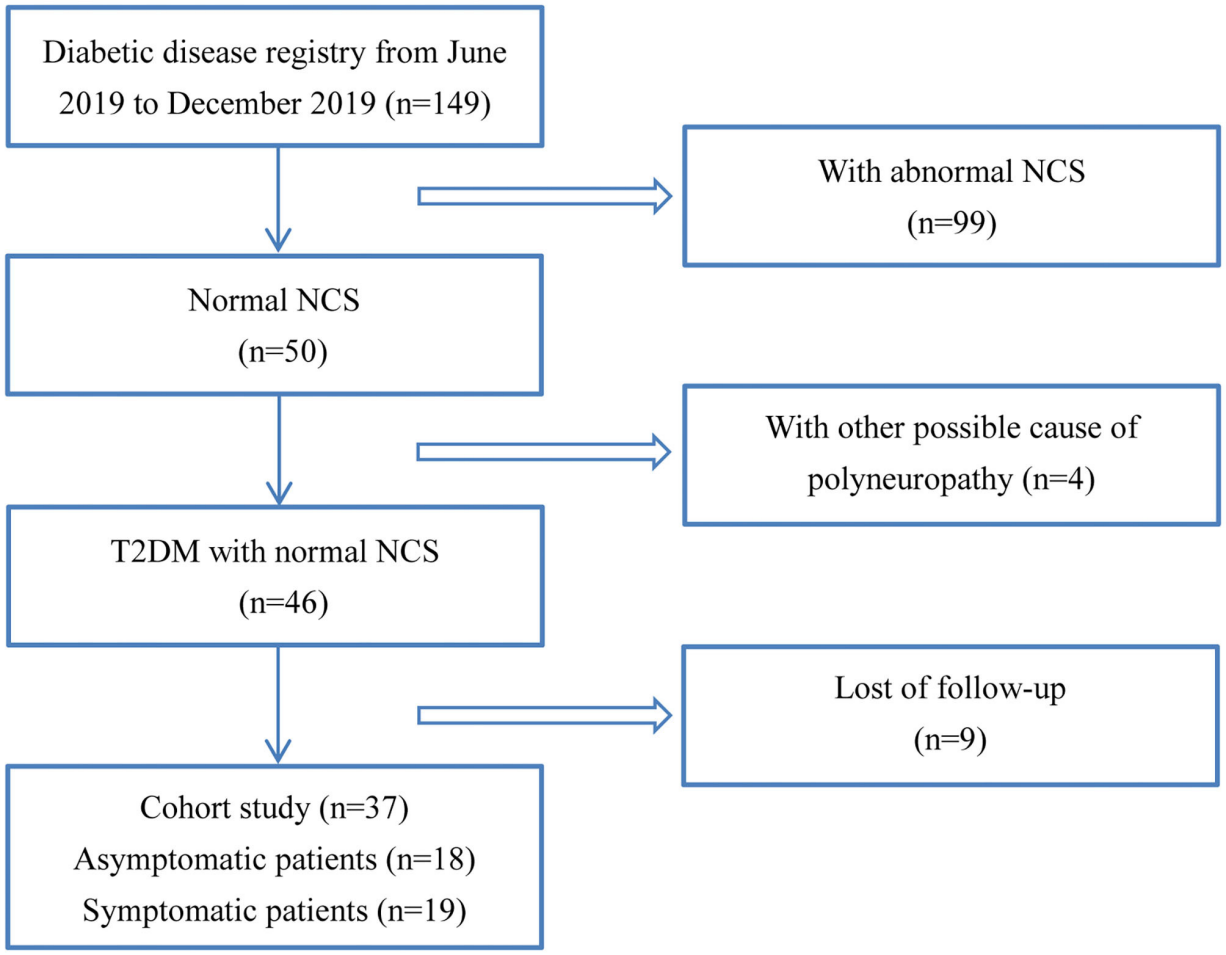
Study flowchart. NCS, nerve conduction studies; DPN, diabetic peripheral neuropathy.

From June 2019 to December 2020, all the in-patients with type II diabetes from the Department of Endocrinology in the First Affiliated Hospital of Sun Yat-sen University underwent NCS, which was a part of the standard practice for the diagnosis of DPN. Thirty-seven patients whose NCS data were in normal range and received follow-up for about 6 months were recruited. All patients underwent a careful interview and neurologic examination including mental status, cranial nerve function, sensory tests for light touch, pin-prick, vibration and joint position sense, muscle strength, balance, gait, coordination, and reflexes. DPN was diagnosed and graded by clinical neurologic assessment and NCS (Pop-Busui et al., 2017). Based on the findings, 18 out of the 37 patients with normal NCS, showing no clinical signs or symptoms of neuropathy, were considered as asymptomatic patients. The remaining 19 patients were defined as symptomatic diabetic neuropathy, which with symptoms and/or signs of neuropathy (symptoms referred to decreased sensation, positive neuropathic sensory symptoms; signs referred to symmetric decrease of distal sensation or unequivocally decreased or absent ankle reflexes) (Pop-Busui et al., 2017). The study flowchart of patient recruitment was shown in Figure 1. The neuropathic symptoms were evaluated on admission and 6 months later, with diabetic neuropathy symptom (DNS) scores that has been previously validated (Meijer et al., 2002). Twenty-two age and height matched healthy participants were included. For diabetic patients and healthy controls, participants with type I diabetes, uremia, chronic alcohol abuse, rheumatoid arthritis, hypothyroidism, nutrition deficiency, malignancy, radiculopathy, or other disorders known to cause polyneuropathies had been excluded.

### Nerve Conduction Study

We measured distal skin temperature in each limb before and after the nerve conduction and F-wave studies to verify that the limb under study remained 32°C or warmer throughout the procedure. The electrodiagnostic studies were performed on Viking EDX electromyography system (Nicolet Biomedical, Madison, WI, USA). Motor nerve conduction studies and F-waves were performed using surface electrodes with a standardized technique (Pan et al., 2014). Motor nerve conduction were examined in the median, ulnar, peroneal, and tibial nerves bilaterally. F-waves were investigated in the median, ulnar and tibial nerves, but not in the peroneal nerves where the F-waves may not occur even in healthy individuals. The active electrodes were placed over the middle of the muscle belly (abductor pollicis brevis, abductor digiti minimi, abductor halluces brevis, and extensor digitorum brevis, respectively) and the reference electrodes were placed on the tendon distally. Sensory nerve conduction was performed in median, ulnar, tibial, peroneal and sural nerves using orthodromic technique. Parameters including distal onset latency for motor nerves, amplitude, and conduction velocity for both motor and sensory nerves, and F-waves occurrence, mean, and minimal latency were analyzed.

### A-Wave Study

The median, ulnar, and tibial nerves were stimulated 70–80 mm proximally to the recording electrode using electrical stimuli of 0.1 ms duration at a frequency of 1 Hz. A 20 supramaximal stimuli were employed to elicit F-waves. The sweep speed was 10 ms/div for leg nerves and 5 ms/div for arm nerves. A-waves were defined according to the following criteria (Rowin and Meriggioli, 2000; Fang et al., 2017): (1) supramaximally elicited reproducible waveforms with amplitudes >0.05 mV, clearly separated from the M-wave; (2) variation of onset latencies <1.5 ms; and (3) occurrence in at least 8 out of 20 stimuli. The late component of the M-wave due to slow conduction in distal nerve branches between cathode and muscle, or repetitive firing of some motor units should be excluded from the A-waves measures. And these can be excluded by stimulating through a second cathode placed 2–3 cm distally over the course of the nerve. The frequency of A-waves in asymptomatic patients, symptomatic patients and healthy controls, differences in clinical characteristics between patients with and without A-waves were analyzed.

### CVD Study

We performed CVD tests based on the antidromic recurrent block of the proximal stimulus (the so-called collision method) (Dalkilic et al., 2015; Koszewicz et al., 2019; Ni et al., 2020) in the median, ulnar, peroneal nerves. Two supramaximal stimuli were delivered subsequently at the distal and proximal stimulation points along the nerves, the two evoked CMAPs were recorded. The interstimulus interval (ISI) was changed according to the distance between the 2 points of stimulations and extended gradually. Initially, ISI was short; therefore, only the first CMAP from the distal point of stimulation was obtained because the action potential from the proximal point of stimulation was blocked by colliding antidromic current. As the ISI was lengthened, the second waveforms of the proximal responses changed so that the amplitudes increased gradually to the same value as the amplitude elicited by distal stimulation. The method allowed estimation of the lower (10%) and upper (90%) quartiles of conduction velocities, as well as the median value (50%). The histograms representing the stepwise increase in cumulative areas of the second CMAPs were calculated on the basis of quartiles of conduction velocities, equal to the relative contribution of the excited fibers with a particular NCV, which was taken to be proportional to the number of fibers (Garssen et al., 2006). In addition, the CVD histogram was intentionally divided into three subgroups based on the value from healthy controls, slow, medium and fast for the ease of interpretation. Since continuous proximal stimulus on tibial nerves did not yield to constant CMAP, tibial nerve was not included for CVD test.

### Statistical Analysis

The Kolmogorov–Smirnov test was used to estimate the normality of the data. The homogeneity was tested using a Levene test. If the data were normally distributed, variables between two groups were compared using the unpaired Student's *t*-test, and a univariate ANOVA was performed for three-group comparisons with a *post hoc* Newman–Keuls test. For data not normally distributed, Mann–Whitney U test was used to compare the means of two groups, and Kruskal–Wallis H test was performed to test the difference in means of three groups. A χ^2^ test was used for categorical data. *P* < 0.05 was considered statistically significant. If not otherwise stated, data were expressed as the mean ± SD. All statistical analyses were conducted using SPSS for windows, version 20 (SPSS, Inc., Chicago, Illinois, USA).

## Results

### Clinical Profile

There were 10 males and 8 females in the asymptomatic group (age 49.5 ± 9.9, range 31–63 years), 11 males and 8 females in the symptomatic group (age 55.7 ± 10.2, range 38–69 years), and 10 males and 12 females in healthy controls (age 47.3 ± 11.4, range 32–75 years). There was no significant difference between the patients and healthy controls. The comparisons of the clinical characteristics between the asymptomatic and symptomatic patients with and without A-waves were shown in Table 1. The disease duration and level of hemoglobin A1c (HbA1c) were comparable between the patients with and without A-waves in each group. The DNS scores did not significantly change during the 6-month follow-up.

**Table 1:** Clinical profile of patients with type II diabetes.

**Parameter**	**Asymptomatic patients**		***P***	**Symptomatic patients**		***P***
	**With A-waves**	**Without A-waves**		**With A-waves**	**Without A-waves**	
Age (years)	46.1 ± 12.1	52.2 ± 8.0	0.22	53.6 ± 11.7	57.4 ± 9.4	0.47
Male:female	6:2	4:6	0.14	6:3	5:5	0.46
Duration of disease (years)	4.5 ± 6.5	5.5 ± 3.6	0.68	9.1 ± 5.6	8.0 ± 6.6	0.71
HbA1c (%)	10.4 ± 1.7	8.9 ± 1.9	0.14	7.3 ± 1.7	8.5 ± 1.9	0.18
DNS score (on admission)	0	0	/	1.0 ± 0.9	0.8 ± 0.7	0.63
DNS score (6 months later)	0	0.25 ± 0.46	/	0.57 ± 0.79	0.67 ± 1.0	0.59

*Values were given as mean ± SD. HbA1c, Hemoglobin A1c; DNS, diabetic neuropathy symptom*.

### The Occurrence of A-Waves

**Figure 2:**
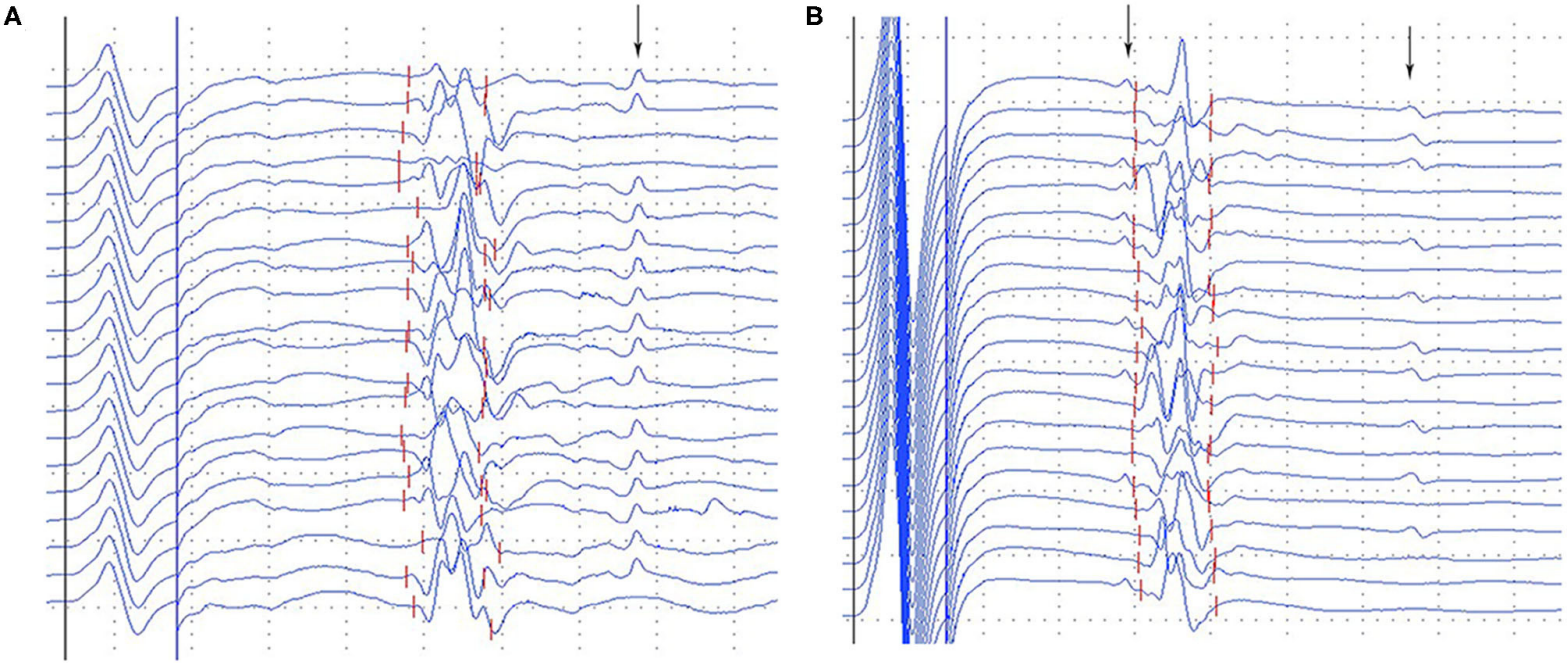
Representative images of A-waves in the diabetic patients. **(A)** Late A-waves following the F-waves with 80% persistence. **(B)** Double A-waves occurring before the F-waves with 45% persistence and after the F-waves with 50% persistence, respectively. The A-waves were marked by an arrow. Dural gain was used; during the M-wave, the gain was 5 mV/div, and during the late responses, the gain was 0.5 mV/div.

A-waves were observed in 45.9% of diabetic patients, 8 out of 18 (44.4%) asymptomatic patients, 9 out of 19 (47.4%) symptomatic patients, and only one single subject (4.5%) among healthy controls, all detected in the tibial nerves. The frequency of A-waves occurring in the asymptomatic patients was similar to that in the symptomatic patients (χ^2^ = 0.032, *P* = 0.858). A-waves were observed between M-waves and F-waves in most patients, after F-waves in a few cases (Figure 2). Most of the A-waves appeared as a single waveform while a few appeared as double -waves (Figure 2).

### Neurophysiological Parameters From Conventional NCS

Asymptomatic patients and symptomatic patients without A-waves showed mildly but significantly reduced motor nerve velocity of the peroneal nerve compared to the patients with A-waves and the healthy controls (Table 2). And the F-wave minimal latency and motor nerve velocity of the tibial nerves in the patients without A-waves were significantly slower than healthy controls. However, the data were identical either between the patients with A-waves and the healthy controls, or between asymptomatic and symptomatic patients (Table 2). The distal latency and amplitudes of CMAPs, mean F-wave latency, as well as the velocity and amplitudes of sensory nerve action potentials were comparable among patient groups and healthy controls. All the data in NCS were in normal range of our laboratory.

**Table 2:** Overview of motor nerve conduction velocity.

**Parameters**	**Asymptomatic patients**		**Symptomatic patients**		**Control**
	**With A-waves**	**Without A-waves**	**With A-waves**	**Without A-waves**	
Median nerve	55.9 ± 2.5	55.2 ± 1.5	56.0 ± 2.1	55.1 ± 2.0	57.1 ± 2.5
Ulnar nerve	56.0 ± 1.9	55.8 ± 1.8	56.4 ± 3.5	55.0 ± 2.3	57.9 ± 3.1
Peroneal nerve	48.1 ± 1.9	46.4 ± 2.1^#^	47.7 ± 1.5	45.9 ± 2.0^#^	49.4 ± 2.1
Tibial nerve	49.2 ± 2.9	48.3 ± 3.6^*^	48.8 ± 4.4	46.8 ± 3.7^*^	51.6 ± 2.4
Tibial F min lantency	44.97 ± 1.35	45.60 ± 3.80^*^	44.86 ± 2.65	46.05 ± 2.78^*^	43.24 ± 2.88

*Values were given as mean ± SD*.

# *P < 0.05 vs. patients with A-waves in the same grading group and healthy controls*.

* *P < 0.05 vs. healthy controls. MCV, motor nerve conductive velocity*.

### Motor Nerve Velocity Distribution in Patients With and Without A-Waves

**Figure 3:**
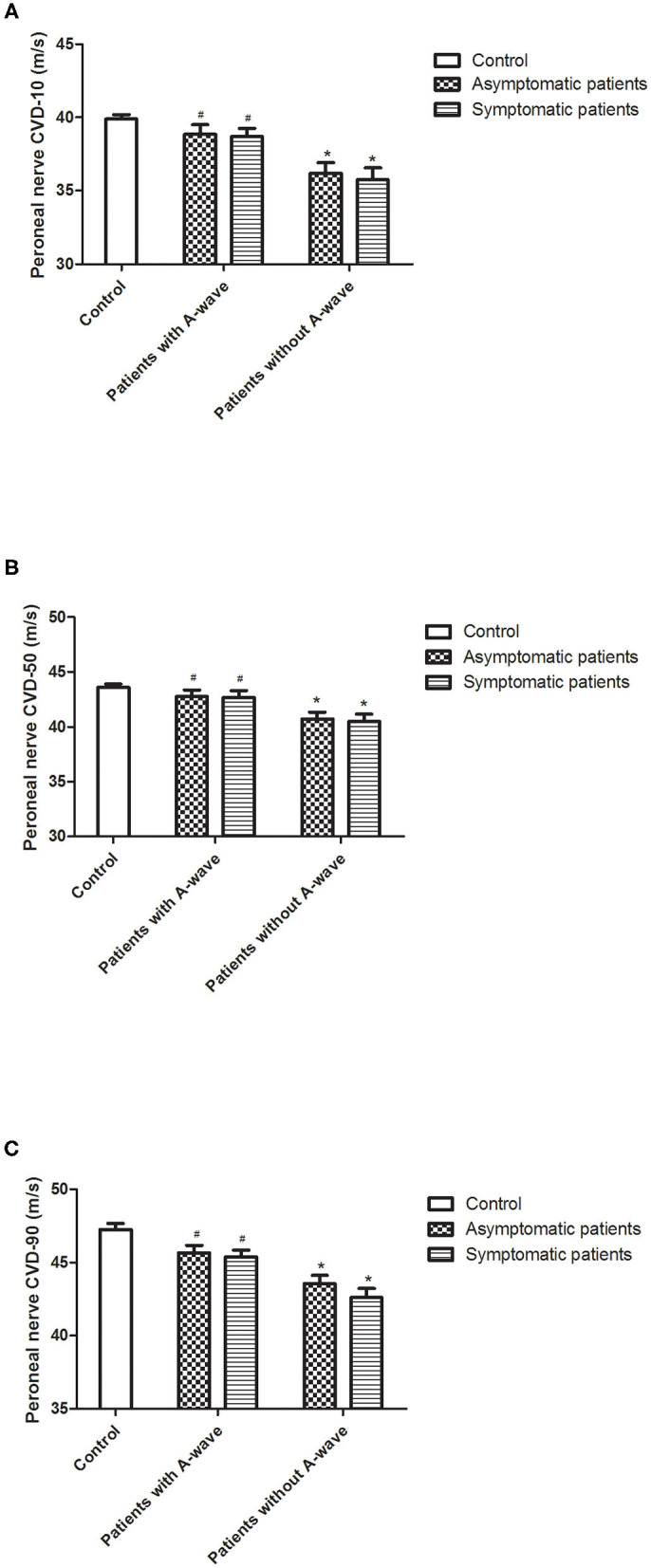
Calculated conduction velocity distribution (CVD) percentiles of peroneal nerves. Results for lower [10%, **(A)**], median [50%, **(B)**], and upper [90%, **(C)**] quartiles of conduction velocities in diabetic patients and healthy controls. All the velocity quartiles were decreased in diabetic patients without A-waves compared to the healthy controls, but relatively reserved in the patients with A-waves. Values were given as mean ± SEM. ^#^*P* < 0.05 vs. patients without A-waves; *P < 0.05 vs. healthy controls.

In diabetic patients with A-waves, motor conduction velocities of the peroneal nerve in all quartiles (10, 50, and 90% of conduction velocities) were significantly faster compared to the patients without A-waves, and showed a trend to be slower than the healthy controls but with no statistical difference (Figure 3). Both asymptomatic and symptomatic groups presented identical velocity in all quartiles (Figure 3) (Supplementary Table 1). The parameters from the median and ulnar nerves were not significantly different either between the patients with and without A-waves, or between the patients and the healthy controls.

**Figure 4:**
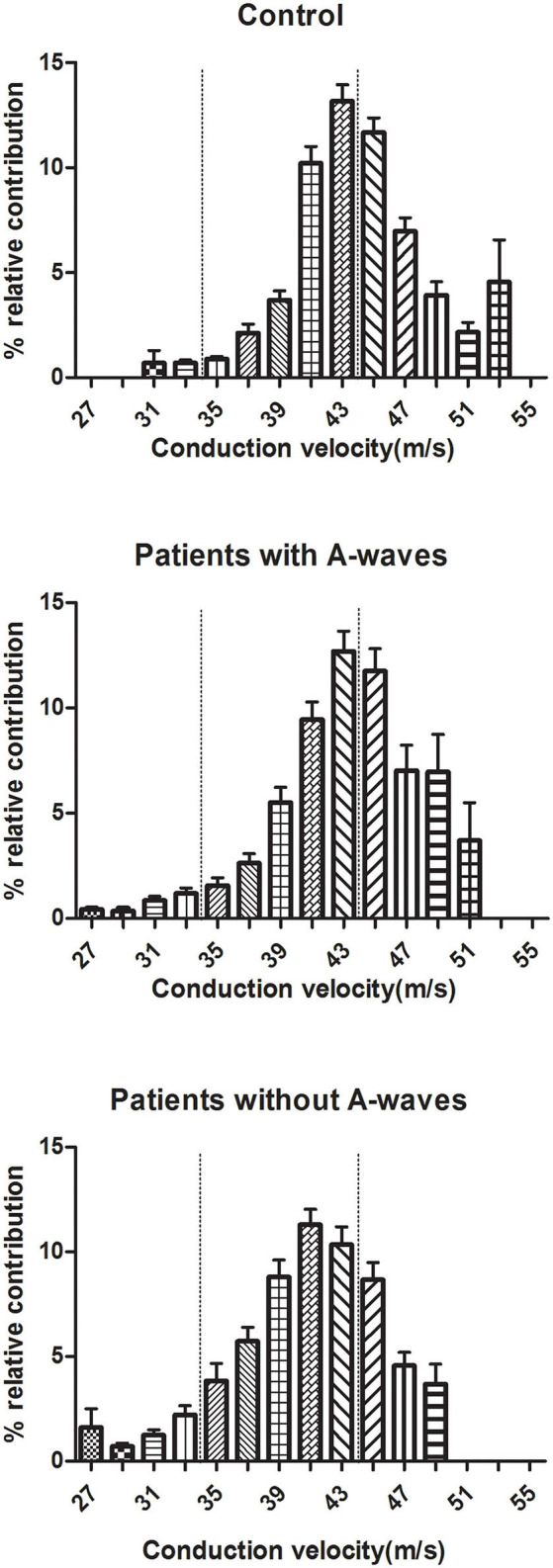
CVD histograms of constituted conduction velocity of peroneal nerves. The histograms represented the stepwise increase in CMAP area, equal to the relative contribution of the excited fibers with a particular NCV. Three subgroups of fibers were designated according to the velocity range of healthy controls, and the borders of slow, medium, fast fibers were shown as dashed lines. Values were given as mean ± SEM.

**Figure 5:**
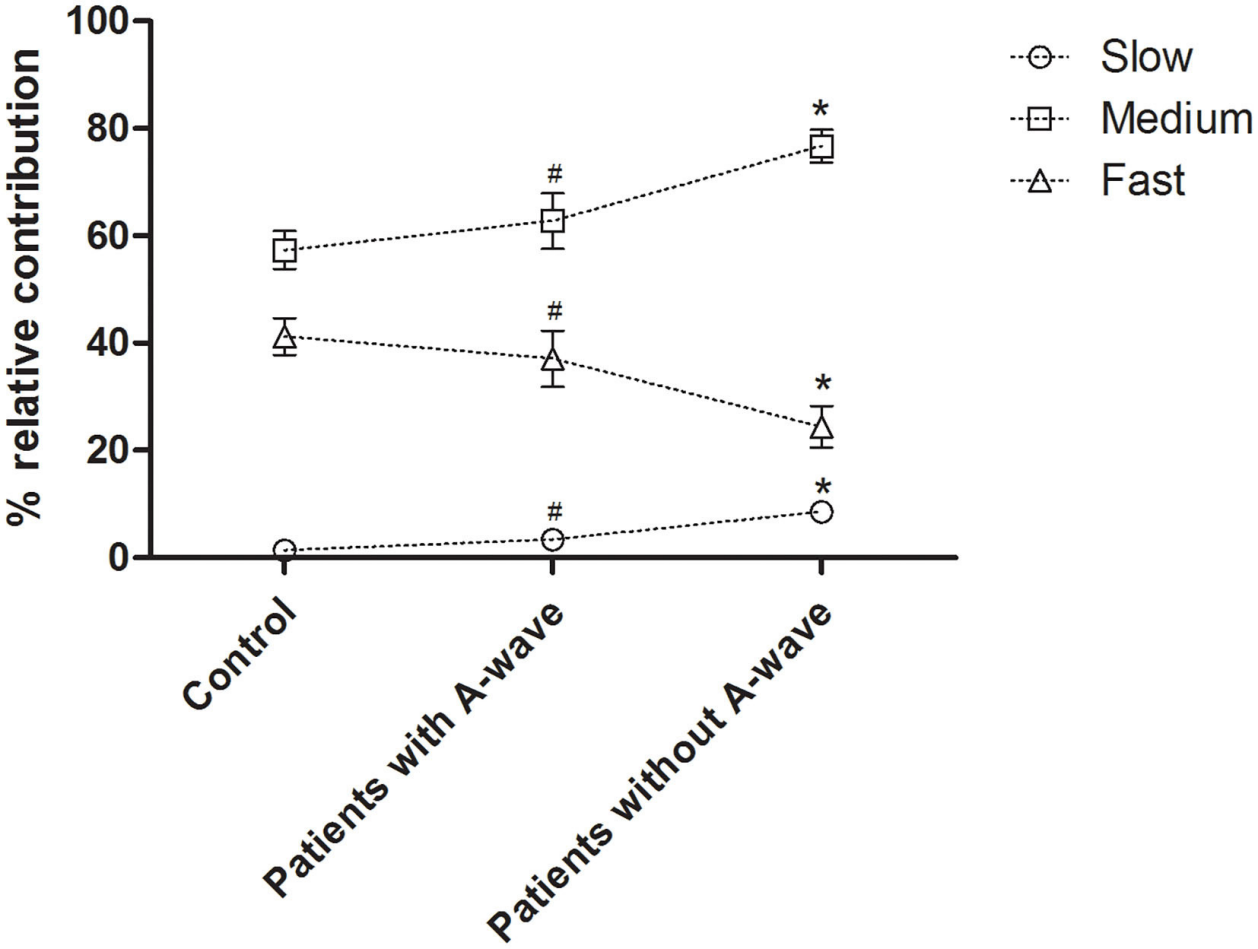
Percent relative contributions of constituted conduction velocity subgroups in peroneal nerves. The contribution of the fast fibers was significantly decreased in diabetic patients without A-waves but not in the patients with A-waves, and the contributions of medium and slow fibers were relatively increased. Slow, 27–35 m/s; medium, 36–44 m/s; fast, 45–53 m/s. Values were given as mean ± SEM. ^#^*P* < 0.05 vs. patients without A-waves; *P < 0.05 vs. healthy controls.

Motor CVD histograms of peroneal nerves were shown in Figure 4. Graphically, the histograms of both healthy controls and diabetic patients with A-waves were shifted to the right side compared to the patients without A-waves, consisting of a larger percentage of faster conducting fibers. To provide a better assessment of motor fiber groups with different conduction velocities, three conduction velocity subgroups were defined according to the velocity range of the healthy controls. The borders in these subgroups were shown on the CVD histograms as dashed lines in Figure 4. The designations and ranges of these subgroups were as follows: slow, 27–35 m/s; medium, 36–44 m/s; fast, 45–53 m/s. For each of these subgroups, relative contributions of motor fibers were recalculated, shown in Figure 5. Since motor conduction velocities were identical in all quartiles between asymptomatic and symptomatic patients as Figure 3 presented, these patients were not separately analyzed in this part. The motor fibers with fast and medium velocities contributed predominantly (more than 40% and nearly 60%, respectively) in the controls, while the contribution of slow fibers was relatively small. The contribution of fast fibers was significantly augmented and that of medium and slow fibers was relatively diminished in diabetic patients with A-waves, compared with patients without A-waves. There was no statistical difference between diabetic patients with A-waves and healthy controls. The data illustrated the loss of fast motor fibers in diabetic patients without A-waves, which was relatively reserved in diabetic patients with A-waves.

## Discussion

In this study, we showed that A-waves could occur in type II diabetic patients when standard NCS retained normal. The patients with A-waves showed faster conduction velocity in all quartiles in the motor peroneal nerves compared to the patients without A-waves, and their histograms were shifted to the right side, consisting of a significantly larger percentage of fast conducting fibers. On the contrary, the patients without A-waves showed a loss of fast motor fibers.

A-waves are compound muscle action potentials that follows the M-waves with a constant morphology and latency. It occurs in healthy individuals on a very low frequency. There are several possible pathophysiologic mechanisms responsible for the generation of A-waves, including demyelination, hyperexcitability of proximal nerve segments, ephaptic transmission from one axon to another, or myoaxonal ephapse (Bischoff, 2002; Jerath and Kimura, 2019). And it is also attributed to the effect of collateral sprouting phenomena within partially denervated muscle and could represent a sign of potential recovery (Havton et al., 2001). Thus A-waves have been considered as a pathological sign of peripheral neuropathy, with generalized and demyelinating origin, particularly common in the early stages of Guillain–Barre' syndrome (Kornhuber et al., 1999; Sartucci et al., 2010), or with axonal or neuronal origin occurring in patients with amyotrophic lateral sclerosis (Fang et al., 2017). A study performed in healthy individuals showed that participants with A-waves appeared to have higher risk to develop future neuropathy, favoring the pathological origin of A-waves (Srotova et al., 2017). Very recently, A-waves were found to occur in 32% DPN patients, mostly in tibial nerves, with unknown physiological significance (Rampello et al., 2019). In the present study, A-waves were also detected in tibial nerves in 45.9% of type II diabetic patients, when standard NCS was still normal. The significantly increased occurrence of A-waves in diabetic patients compared to that in the normal controls suggested that A-waves be an early sign of pathological process, which however, did not necessarily imply worse nerve function.

The peripheral nerves consist of axons of different diameter, unmyelinated and myelinated with different thicknesses of the myelin sheaths. The conduction velocity depends on both the diameter of the axons and the thickness of the myelin (Ugrenović et al., 2016; Koszewicz et al., 2019). The collision method of CVD allowed us to explore virtually the whole spectrum of fibers forming the group A nerve fibers connecting alpha motoneurons and extrafusal muscle fibers including large and small myelinated fibers (Kiziltan et al., 2007; Ni et al., 2020). Previous studies (Dorfman et al., 1983; Bertora et al., 1998) have confirmed the sensitivity of CVD in detecting early neuropathy in diabetic patients. It is also known from experimental nerve resection studies and from studies in Guillain–Barre' patients that CVD may be found impaired alongside normal conventional NCS (Garssen et al., 2006; Kiziltan et al., 2007; Tuncer et al., 2011). Here with the collision method, we could find mild neuropathy in the diabetic patients who presented normal conventional NCS, and could assess the detailed motor nerve function in these patients with and without A-waves, thus determine at least part of the physiologic significance of A-waves. The velocities of the determined CVD percentiles (10, 50, and 90%) were all significantly reduced in the peroneal nerve only in type II diabetes patients without A-waves, not in patients with A-waves, compared to that in the healthy controls. The difference was not significant in ulnar and median nerves. These results supported a length-dependent neuropathy, where nerves in lower limbs were more vulnerable than those in upper limbs (Bae and Kim, 2007; Gylfadottir et al., 2019; Ziegler, 2020). The patients with A-waves possessed significantly faster motor fibers of peroneal nerves in all velocity quartiles compared to the patients without A-waves. Fast motor fibers were significantly lost in patients without A-waves compared to the healthy controls, thus the relative contributions of medium and slow motor fibers were augmented despite of the susceptibility of small nerve fibers with slow conduction velocities in diabetes. The loss of faster-conducting fibers was in line with a previous study in diabetic rats (Tuncer et al., 2011). However, fast motor fibers were functionally reserved in patients with A-waves, thus contributed more to the CMAP, shifting the CVD histograms to the right side. Consistent with these results, it was reported that peripheral nerve pathology in diabetic patients is characterized by progressive nerve fiber loss with pan-modal fiber size pattern (Dyck and Giannini, 1996; Yagihashi et al., 2007). It has been recently shown that the involvement of large fibers in early stages of diabetic patients was related to reduced nerve conduction velocity or decreased vibration threshold. And as an early sign, there is a loss of intra-epidermal nerve fibers that can be detected by immunohistochemistry (Dyck et al., 2000; Polydefkis et al., 2003). If so, CVD results should be a better measure for detecting early conduction changes, as studies of longer nerves are more sensitive for study of diffuse processes. Because the relation between the diameter of a nerve fiber and conduction velocity of action potential is one of the strongest anatomical and function relations in neurophysiology and neuroscience (Hartline and Colman, 2007), the results supported that the occurrence of A-waves may imply the reserved motor nerve function of large-diameter fibers. Likewise, a recent study of patients with amyotrophic lateral sclerosis has found that A-waves correlated with slower disease progression rate and degeneration process (Fang et al., 2017). In our study, DNS score did not yet show significant changes during the short-term follow-up between the patients with and without A-waves, but future long-term follow-up may make some difference.

At present, the precise mechanisms of supramaximally elicited A-waves in diabetes mellitus with normal NCS is still unclarified. It is known that reinnervation occurs in early DPN, confirmed in previous studies by pathology wherein nerve fiber sprouts among fascicles along with patchy fiber degeneration (Kennedy and Zochodne, 2005; Andreassen et al., 2014; Khoshnoodi et al., 2019), and by single fiber electromyography wherein both jitter value and muscle fiber density were increased (Bril et al., 1996; Sanders et al., 2019). Additionally, it was reported that innervation ratio for motor nerve fibers with fast conduction velocity is larger than those with slow conduction velocity (Burke and Tsairis, 1973). Whether A-waves could originate from reinnervation of large myelinated motor fibers of peripheral nerves, which compensated the motor nerve function, remain to be determined.

There are some limitations of the present study. First, a technical issue related to the refractory period may underestimate the conduction velocity obtained in the collision test (Ni et al., 2020). Because all the groups of subjects were studied in the same way and the results from CVD test were also supported by data of motor nerve velocity and minimal F-wave latency, we believe that the combinative use of neurophysiological methods gave valuable information to evaluate motor nerve function in the present study. Second, A-waves were found mainly in tibial nerves, but CVD study was not performed in the same nerve for a technical reason that repetitive proximal stimulus of tibial nerves was not reliable. Since DPN is typically a length-dependent sensorimotor polyneuropathy, CVD study in peroneal nerves can serve as a reasonable alternative to reflect the peripheral nerve function.

## Conclusion

In conclusion, NCS could detect high occurrence of A-waves in type II diabetic patients as early as when all the routine conductive parameters retained in normal range. Though length-dependent nerve degeneration was an ongoing process, the patients with A-waves was characterized with faster conductive motor fibers in all velocity spectrum, and relative reservation of fast fibers, compared to the patients without A-waves. The data suggested that A-waves be a pathological sign in early DPN, as well as a sign of rescued motor nerve function of large-diameter fibers. Future studies in pathology and neurophysiology are needed to clarify the mechanisms underlying the functional reservation of patients with A-waves and their long-term change of nerve conductive function.

## Data Availability Statement

The original contributions presented in the study are included in the article/Supplementary Material, further inquiries can be directed to the corresponding author.

## Ethics Statement

The studies involving human participants were reviewed and approved by the Ethics Committee of the First Affiliated Hospital of Sun Yat-Sen University, Guangdong, China. The patients/participants provided their written informed consent to participate in this study. Written informed consent was obtained from the individual(s) for the publication of any potentially identifiable images or data included in this article.

## Author Contributions

The authors declare that they have each made substantial contributions to the conception, acquisition, analysis, and interpretation of the manuscript. All authors have critically revised the manuscript for intellectual content and have given their approval for the final version to be published.

## Conflict of Interest

The authors declare that the research was conducted in the absence of any commercial or financial relationships that could be construed as a potential conflict of interest.
