# Supplementary material for: The Physiological Significance of A-Waves in Early Diabetic Neuropathy: Assessment of Motor Nerve Fibers by Neurophysiological Techniques
Source: Front Syst Neurosci. 2021 Jan 28;15:633915. doi: 10.3389/fnsys.2021.633915 (PMC7876338; doi:10.3389/fnsys.2021.633915)
Supplement: Supplementary file 1 [file Table_1.DOCX]

Supplementary Table 1. Calculated conduction velocity distribution percentiles of peroneal nerves.

| CVD percentiles (m/s) | Asymptomatic patients | | | Symptomatic patients | | | Control | |
| --- | --- | --- | --- | --- | --- | --- | --- | --- |
|  | With A-waves | | Without A-waves | | With A-waves | Without A-waves | |  |
| CVD-10 | | 38.84±1.97^#^ | 36.18±2.96^*^ | | 38.68±1.58^#^ | 35.76±2.14^*^ | | 39.88±1.62 |
| CVD-50 | | 42.77±1.89^#^ | 40.72±2.57^*^ | | 42.67±1.90^#^ | 40.48±2.03^*^ | | 43.59±1.61 |
| CVD-90 | | 45.65±1.70^#^ | 43.56±2.09^*^ | | 45.37±1.25^#^ | 42.60±1.38^*^ | | 47.24±2.08 |

Values were given as mean ± SD. ^#^*P*<0.05 vs. patients without A-waves; **P*<0.05 vs. healthy controls. CVD, conduction velocity distribution.
